# Dietary context and ethanol-related injury in preclinical models: A contemporary systematic review of predominantly obesogenic diets and liver outcomes

**DOI:** 10.1371/journal.pone.0352561

**Published:** 2026-10-21

**Authors:** Worship O. Agbonifo, Joseph Chimezie, Zainab A. Amoo, Chidinma Chukwukaeme, Hope O. Francis, Mercy O. Awoleye, Temitope G. Adedeji

**Affiliations:** 1 Epigenetics and Molecular Biology Laboratory, Federal University of Technology, Akure, Nigeria; 2 Department of Physiology, School of Basic Medical Sciences, Federal University of Technology, Akure, Nigeria; 3 Biochemical and Molecular Pharmacology and Toxicology Laboratory, Department of Biochemistry, School of Life Sciences, Federal University of Technology, Akure, Nigeria; 4 Department of Physiology, Michigan State University, East Lansing, Michigan, United States of America; 5 Department of Physiology, Nnamdi Azikiwe University Awka, Awka, Nigeria; Anhui University of Chinese Medicine, CHINA

## Abstract

Diet composition is an important food-derived exposure that may influence alcohol-related tissue injury. However, evidence on how specific dietary contexts modify ethanol-induced injury and its underlying biological mechanisms in controlled preclinical models remains fragmented. This systematic review examined whether defined dietary patterns modify ethanol-related injury in controlled mammalian studies. Following PRISMA 2020, PubMed, Embase, and Scopus were searched for studies published from January 2021 to January 15, 2026. Eligible studies were controlled in vivo mammalian experiments comparing ethanol exposure under defined dietary patterns. Evidence was narratively synthesized by diet type, organ system, and mechanism; random-effects meta-analyses and prespecified interaction analyses were conducted when sufficiently comparable data were available. Twenty-seven studies met the inclusion criteria (24 mouse and three rat studies); 18 used male-only animals, most examined high-fat or Western-type diets, and liver outcomes predominated. Evidence for non-obesogenic diets was limited to one ketogenic-diet study. In acute high-fat-diet models, adding ethanol increased serum ALT (SMD 2.31, 95% CI 1.46–3.16), AST (SMD 1.18, 95% CI 0.54–1.81), and hepatic triglycerides (SMD 2.06, 95% CI 0.96–3.17). Acute interaction analysis identified dietary effect modification only for ALT (MD 74.56, 95% CI 59.03–90.10), while chronic analyses showed a negative interaction for hepatic triglycerides (MD −0.048, 95% CI −0.092 to −0.004; P = 0.03). Extrahepatic, microbiome, and multi-omics findings were sparse and heterogeneous. High-fat and Western-type diets generally exacerbated ethanol-induced hepatic injury, whereas evidence for other dietary patterns remains limited.

## Introduction

Alcohol remains a major global determinant of morbidity and premature mortality. In 2019, alcohol use was associated with an estimated 2.6 million deaths and 116 million disability-adjusted life-years (DALYs), representing 4.7% of all deaths and 4.6% of total DALYs worldwide [1]. This burden is not evenly distributed and comparative global estimates continue to show marked regional heterogeneity, with particularly high alcohol-attributable burden in parts of Europe and substantial burden across sub-Saharan Africa [1]. At the mechanistic level, ethanol, the main component of alcoholic beverages, disrupts cellular redox homeostasis during its metabolism by increasing the hepatic NADH/NAD⁺ ratio [2]. In hepatocytes, this redox shift impairs fatty-acid β-oxidation and promotes hepatic triglyceride accumulation. Excessive alcohol exposure also increases reactive oxygen species production, depletes antioxidant defences, enhances lipid peroxidation, and activates inflammatory signalling cascades [2]. Importantly, these effects are not confined to the liver, as alcohol disrupts gut epithelial barrier integrity, alters the composition of the intestinal microbiome, and facilitates the translocation of bacterial products such as lipopolysaccharide, thereby linking gut dysfunction to inflammatory signalling in the liver and other tissues [3]. Beyond the liver and gut, chronic alcohol exposure also affects multiple extrahepatic organs, highlighting the multisystem nature of alcohol-related injury [4]. It can promote neuroinflammation and neurodegeneration, contribute to cardiovascular disorders including hypertension, arrhythmias, and stroke, drive pancreatic injury, impair pulmonary defences, and disrupt skeletal muscle protein synthesis [4–8].

Diet is another major determinant of population health, although its effects vary substantially depending on overall dietary pattern [9]. In 2019, dietary risk factors were associated with 7.9 million deaths and 187.7 million DALYs from non-communicable diseases worldwide, with high sodium intake and low intakes of whole grains and fruits ranking among the leading contributors [10]. At the same time, dietary patterns in many low- and middle-income (LMIC) settings have shifted toward greater intake of processed foods, alongside a substantial global expansion in ultra-processed foods and rapid growth in their availability and consumption across LMICs [11,12]. The Western diet, characterized by high intake of saturated fats, refined carbohydrates, red and processed meats and sugar-sweetened beverages, has been seen to lower antioxidant status, increase reactive oxygen species and promote systemic inflammation. This pattern raises LDL cholesterol, lowers HDL cholesterol and increases the risk of cardiometabolic diseases, cancer and neurodegenerative disorders [13]. In contrast, the Mediterranean diet lays emphasis on fruits, vegetables, legumes, whole grains, nuts, fish and extra-virgin olive oil and is associated with reductions in chronic disease, neurologic disorders, cardiometabolic risk, cancer, diabetes, liver dysfunction and mortality [14]. The ketogenic diet is a very low-carbohydrate, high-fat regimen originally used for epilepsy. It increases circulating ketone bodies and may reduce insulin levels and suppress appetite but can raise LDL and total cholesterol [15].

Against this backdrop, the biological consequences of alcohol use are unlikely to depend on ethanol exposure alone, but also on the broader dietary environment in which drinking occurs [16,17]. Emerging evidence suggests that alcohol and adverse dietary exposures converge on several shared pathogenic pathways, including disordered lipid metabolism, oxidative stress, intestinal dysbiosis, epithelial barrier disruption, endotoxemia and chronic inflammation, thereby amplifying injury along the gut-liver axis and potentially influencing extrahepatic organs through systemic and gut-brain signalling [18–22]. Given this complexity, the interaction between diet and alcohol metabolism and toxicity is best understood by conceptualising diet as an overall pattern rather than as isolated nutrients, since this more accurately reflects real-world exposures [23].

While epidemiological studies show associations between diet, alcohol consumption, and health outcomes, they are limited in causal inference and mechanistic resolution [24]. Preclinical models offer a controlled environment for examining how nutritional context affects the biological effects of alcohol [25,26]. This level of control makes it possible to investigate causal relationships and uncover molecular pathways that are difficult to study in humans, including immunological signalling, oxidative stress, and metabolic dysregulation [26–28]. Such mechanistic evidence is essential for identifying therapeutic targets and informing translational strategies for prevention and intervention [29].

Despite growing evidence that dietary patterns modulate alcohol‑induced injury, existing research remains fragmented. Most animal models focus on the Western/high‑fat diet, yet human populations follow diverse diets including Mediterranean, ketogenic, vegetarian and mixed patterns. Comparative mechanistic data across these patterns are scarce. Furthermore, studies also target specific organs of interest without evaluating systemic interactions that involve gut microbiota, immune signalling, neuroendocrine pathways, mitochondrial function or epigenetic modifications. Therefore, this systematic review aims to identify preclinical studies combining ethanol exposure with defined dietary patterns, extracting mechanistic endpoints and comparing outcomes across multiple body systems.

## Methods

### Review design and reporting framework

This systematic review was designed as a time-bounded synthesis of contemporary preclinical evidence rather than a historical census of diet-ethanol research and was conducted and reported in accordance with PRISMA 2020 guidelines [30]. The evidence window began on 1 January 2021 to capture the recent generation of metabolically contextualized experimental models and spans the period leading to and following the 2023 multisociety formalization of the metabolic dysfunction-associated steatotic liver disease (MASLD) and metabolic dysfunction and alcohol-associated liver disease (MetALD) framework [31].

### Eligibility criteria

Eligibility criteria for study selection were defined a priori according to the PECOS framework and are summarised in Table 1 [32]. Studies were eligible if they were controlled preclinical experimental studies evaluating the mechanistic effects of experimentally administered ethanol exposure in the context of experimentally defined dietary patterns, with an explicit comparator group (non-ethanol and/or control diet). Eligible studies used in vivo animal models (rodent or non-rodent mammalian models). Studies were excluded if they were clinical/human studies, observational-only animal studies without experimental manipulation, or if they lacked sufficient dietary description to permit classification by dietary pattern or composition. Studies without clearly defined ethanol exposure (e.g., unclear route/dose/pattern) or without an appropriate comparator group were also excluded.

**Table 1:** PECOS inclusion and exclusion criteria.

Domain	Inclusion Criteria	Exclusion Criteria
Population	In vivo preclinical experimental animal models, including rodents and non-rodent mammalian models	Human/clinical studies; in vitro/ex vivo-only studies, non-mammalian models.
Exposure 1: Ethanol	Experimentally administered ethanol/alcohol exposure, including Lieber-DeCarli ethanol liquid diets, intragastric infusion, voluntary drinking, gavage, vapour exposure, and ethanol in drinking water	No ethanol exposure
Exposure 2: Dietary Patterns	Experimentally defined complete diets enabling classification (Western-type, ketogenic, Mediterranean-like, high-fat, high-fructose, high-sucrose, low-/high-protein, etc.)	Diet not described sufficiently to classify pattern/composition
Comparator	Non-ethanol controls and/or control diet groups with otherwise comparable conditions	No comparator group; incomparable controls
Outcome	mechanistic endpoints across organ systems (oxidative stress/redox, inflammation/immune signalling, mitochondrial function/lipid metabolism, barrier integrity/microbiome signalling, neuroendocrine/stress pathways, epigenetic/transcriptomic/proteomic/metabolomic outcomes).	Outcomes unrelated to alcohol-diet mechanisms
Study Design	Controlled preclinical experimental studies with defined exposures and comparator groups	Observational-only animal studies without experimental manipulation, Reviews, Editorials, and Book Chapters

### Information sources

The electronic databases PubMed, Scopus, and Embase were searched for studies published between 1 January 2021 and 15 January 2026, and each database was last searched on 15 January 2026. In addition, the reference lists of included studies were screened to identify any further eligible studies published within the same date range.

### Search strategy

The database searches combined two core concept blocks:

(1) ethanol or alcohol exposure and
(2) dietary pattern or composition.

Synonymous terms within each block were combined using the Boolean operator “OR,” and the two blocks were combined using “AND.” Terms were searched in the title and abstract fields in PubMed, the title, abstract, and keyword fields in Embase, and the title, abstract, and keyword fields in Scopus. The syntax was adapted to the requirements of each database.

The predefined publication window was implemented using database-specific publication-date filters. Organ-system and mechanistic terms were were assessed during study screening to preserve the sensitivity of the search. Complete search strings and all filters applied in each database are reported in S1 Appendix in S1 File.

### Selection process

All records retrieved from the searches were imported into Silvi^®^ Version 1.8.8 (http://app.silvi.ai) accessed 23/01/2026 for deduplication and screening management. Titles and abstracts were screened independently by two reviewers against the eligibility criteria, followed by full-text assessment of potentially relevant reports. Reasons for exclusion at the full-text stage were recorded. Silvi AI was used to support record management and screening workflow, but study eligibility decisions were made by the reviewers.

### Data collection process

Data were extracted from full-text reports using a standardised, piloted data-extraction form developed to align with the review question and PECOS criteria. Two reviewers independently extracted data from each included study/report; discrepancies were resolved through discussion, with a third reviewer adjudicating when consensus could not be reached. Where numerical outcome data were presented only graphically, values were digitised from figures using WebPlotDigitizer (version 5.2; accessed 20/02/2026), a web-based tool for extracting numerical data from chart images [33]. When individual animal-level observations were visible, each observation was digitised and group means and standard deviations were reconstructed directly. The corresponding number of visible observations was used as the group sample size after confirmation against the study report. For graphs presenting only summary estimates, the definition of the error bars was verified from the figure legend or accompanying text before extraction; values reported as standard errors of the mean were converted to standard deviations. Reconstructed summary statistics, group sample sizes and any transformations were recorded in the supplementary data sheet (S1 Dataset in S1 File).

### Data items

Data were extracted at both the study level and the outcome level. Study-level variables included publication year, authors, study title, experimental model, sex, baseline weight, sample size per group, age at baseline, comparator group, duration of exposure, dietary exposure type, ethanol frequency, diet and ethanol composition details, ethanol exposure dose, and additional study notes relevant to interpretation.

Outcome-level data were extracted for all reported outcomes compatible with the pre-specified mechanistic domains, across all reported tissues or organ systems, time points, and analyses where extractable. For each outcome, the following information was recorded: tissue or organ system, outcome domain, specific biomarker or endpoint, assay or method used, time point assessed, direction of effect in the diet-ethanol group, numerical result or summary, statistical significance, and author interpretation. The complete extracted numerical dataset, including digitized and derived values and the analysis-ready inputs used in RevMan, is provided in S1 Dataset in S1 File.

For synthesis, individual outcomes were subsequently grouped into broader mechanistic domains, including oxidative stress/redox signalling, inflammation/immune signalling, mitochondrial function and lipid metabolism, barrier integrity and microbiome-related signalling, neuroendocrine/stress pathways, and epigenetic/multi-omics outcomes. Secondary outcome domains included organ phenotypes relevant to alcohol-diet interactions, such as steatosis, inflammation scores accompanied by mechanistic readouts, gut permeability phenotypes, neuroinflammation, insulin resistance/glucose homeostasis, and cardiometabolic markers.

### Study risk of bias assessment

Risk of bias was assessed at the study level using a tool appropriate for in vivo animal intervention studies (SYRCLE Risk of Bias tool) [34]. The assessment covered the domains of sequence generation, baseline characteristics, allocation concealment, random housing, blinding of caregivers and investigators, random outcome assessment, blinding of outcome assessors, incomplete outcome data, selective outcome reporting, and other sources of bias. Domain-level judgments were made using the tool’s signalling questions and the information reported in the full-text articles. Disagreements were resolved by discussion and consensus. The results of the assessments were summarised across studies, incorporated into the interpretation of the evidence, and visualised using ROBVIS web application (accessed 21/02/2026) [35].

### Effect measures

For quantitative synthesis of continuous outcomes, effect estimates were expressed as mean differences (MDs) when outcomes were measured on the same scale and as standardised mean differences (SMDs) when conceptually similar outcomes were reported using different scales. All pooled estimates were presented with 95% confidence intervals (CIs). For formal interaction analyses in studies with complete 2 × 2 designs, effects were expressed as unstandardized difference-in-differences mean differences when the outcome was reported in a common unit across studies. All pooled estimates were presented with 95% confidence intervals.

### Synthesis methods

Findings from the included studies were synthesised using both narrative synthesis and, where appropriate, meta-analysis. A narrative approach was used to summarise study characteristics and findings across experimental model, dietary exposure type, ethanol exposure pattern and dose, comparator group, tissue or organ system, outcome domain, and time point assessed. Outcomes were first considered as reported in the individual studies and then grouped into broader mechanistic domains to support structured comparison across studies.

Where studies were sufficiently comparable in terms of animal model, dietary exposure, ethanol exposure paradigm, comparator condition, outcome construct, and timing of assessment, quantitative synthesis was undertaken using Review Manager (RevMan, version 10.1.1; accessed 21/02/2026) [36]. For continuous outcomes, pooled effect estimates were calculated as mean differences when outcomes were measured on the same scale, or standardised mean differences when similar constructs were measured using different assays or scales, each with 95% confidence intervals. Statistical heterogeneity was assessed using the chi-square test and quantified using the I² statistic. Where sufficient data were available, potential sources of heterogeneity were explored through subgroup analyses based on factors such as dietary pattern, ethanol exposure model, tissue or organ system, and species. Random-effects meta-analysis was used to account for anticipated between-study variation in animal models and exposure protocols. Between-study variance was estimated using the Restricted Maximum-Likelihood (REML) method, and summary-effect confidence intervals were calculated using the Wald-type method. Where quantitative pooling was not appropriate because of substantial heterogeneity or insufficient comparable data, findings were presented narratively.

Furthermore, in studies with a complete 2 × 2 factorial structure comprising control, ethanol-only, diet-only, and diet-plus-ethanol groups, we performed a prespecified interaction analysis to assess whether dietary context modified the effect of ethanol on the outcome of interest. In keeping with factorial-trial principles, interaction was evaluated directly rather than inferred from separate main-effect comparisons [37]. For continuous outcomes measured on a common scale, the study-specific interaction effect was calculated as a difference-in-differences:


$$\begin{array}{c} \left(Diet\;EtOH-Diet)-(EtOH-Control\right) \end{array}$$

Assuming independent study arms, the variance of the interaction estimate was calculated as the sum of the four group-specific variances and the standard error was obtained as the square root of this variance as shown below.


$$\begin{array}{c} V(I)=\frac{{SD}_{Diet\;EtOH}^{\text{2}}}{n_{Diet\;EtOH}}+\frac{{SD}_{Diet}^{\text{2}}}{n_{Diet}}+\frac{{SD}_{EtOH}^{\text{2}}}{n_{EtOH}}+\frac{{SD}_{Control}^{\text{2}}}{n_{Control}} \end{array}$$

$$\begin{array}{c} SE(I)=\sqrt{V(I)} \end{array}$$

Study-specific interaction estimates and their standard errors were then pooled using the generic inverse-variance method in RevMan Web under a Random-effects model [38]. Between-study variance was estimated using the Restricted Maximum-Likelihood (REML) method, and summary-effect confidence intervals were calculated using the Wald-type method. Studies lacking one or more of the four required groups were not eligible for formal interaction meta-analysis but remained eligible for pairwise meta-analysis or narrative synthesis, as appropriate.

## Results

### Study selection

The study selection process is shown in Fig 1. In total, 2,521 records were identified through database searching. After removal of duplicates, 2,270 records underwent title and abstract screening, of which 190 reports were retrieved for full-text assessment. Following full-text review, 27 studies met the eligibility criteria and were included in the review. The main reasons for exclusion at the screening and full-text stages are presented in Fig 1.

**Fig 1:**
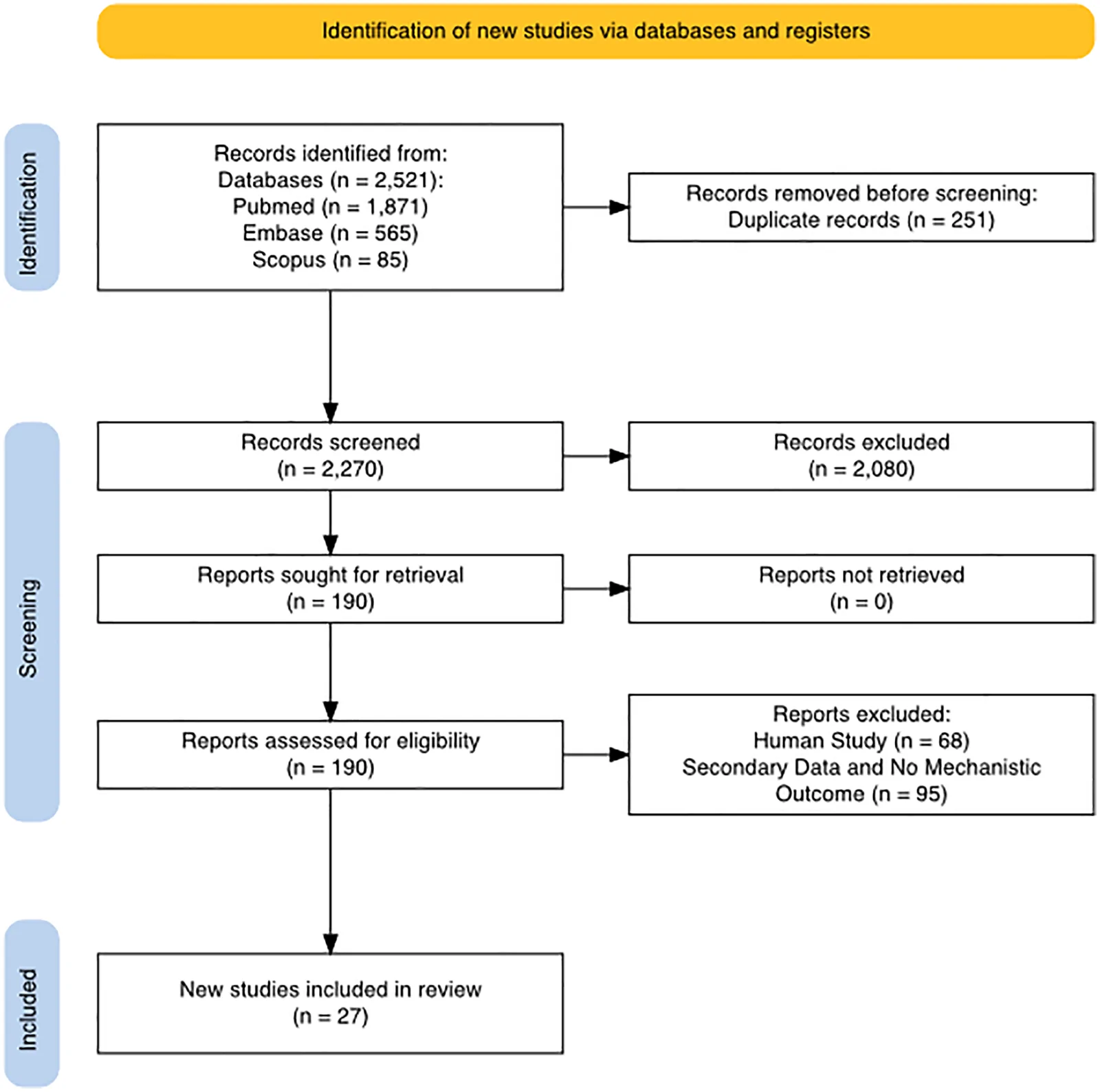
PRISMA flow diagram of study identification, screening, eligibility assessment, and inclusion. Flow diagram showing database records retrieved, duplicates removed, records screened, full-text reports assessed, reasons for exclusion, and the final number of studies included in the systematic review.

### Study characteristics

Characteristics of the included studies are summarised in Table 2, with full study-level details provided in S1 Table in S1 File. The 27 included studies comprised 24 mouse studies and 3 rat studies. Sex distribution was skewed toward males: 18 studies used male-only animals, 7 included both sexes, and 2 were conducted in females only.

**Table 2:** Characteristics of included preclinical studies evaluating the mechanistic effects of dietary patterns on ethanol-induced injury.

Study	Model/sex	Dietary pattern	Ethanol regimen	Duration	Primary mechanistic focus
(Alwahsh et al., 2025) [39]	Rat; male	Lieber-DeCarli HFD with fructose	30% kcal ethanol continuously	4-8 weeks (Chronic)	Liver injury, steatohepatitis, and iron homeostasis
(Antony et al., 2024) [40]	Mouse; female	17% fat, cholesterol/cholate diet	Drinking-water ethanol escalated to 5%	9 weeks (Chronic)	Liver membrane proteome, fibrosis
(Deng et al., 2025) [41]	Mouse; male	3-day HFD (60% kcal fat)	Single 5 g/kg gavage	9 h endpoint (Acute)	Acute liver injury, oxidative stress, and pyroptosis
(Fletcher et al., 2025) [42]	Mouse; both sexes	High-fat, high-sucrose diet	20% v/v in drinking water	16 weeks of alcohol within a 32-week diet study (Chronic)	Ischemic skeletal-muscle mitochondrial dysfunction
(Fu et al., 2025) [43]	Mouse; male	HFD + fructose in ApoE-/- model	1% or 5% drinking-water ethanol	14 weeks (Chronic)	Plaque vulnerability, lipids, aortic transcriptomics
(Gao et al., 2025) [44]	Mouse; male	LFD or HFD (60 kcal% fat)	10% v/v drinking-water ethanol	12 weeks (Chronic)	Liver phenotype, gut microbiota, faecal metabolomics
(González-Portilla et al., 2021) [45]	Mouse; male	Standard vs intermittent/continuous HFD	Adolescent binge injections	2 weeks + follow-up (Mixed)	Striatal cytokines and later ethanol self-administration
(Ismaeel et al., 2022) [46]	Mouse; both sexes	High-fat or low-fat Lieber-DeCarli	Chronic liquid-diet ethanol	6 weeks (Chronic)	Skeletal-muscle mitochondrial dysfunction, redox
(Kim et al., 2021) [47]	Rat; male	20% or 40% fat diets	Daily gastric gavage with ethanol	8 weeks (Chronic)	Liver steatosis, ER stress, apoptosis, and fibrosis signalling
(Liang et al., 2023) [48]	Mouse; male	Long-term HFD	2%, 10%, or 20% ethanol in water	4-39 weeks (Chronic)	Time-dependent miR-29a, histology, fibrosis
(Nataraj et al., 2025a) [49]	Mouse; female	Western diet alcohol (WDA)	Ramped drinking-water ethanol to 20%	8-16 weeks (Chronic)	Hepatocyte Kdm5b, macrophage phenotype, fibrosis
(Nataraj et al., 2025b) [50]	Mouse; male	WDA	Ramped drinking-water ethanol to 20%	12-18 weeks (Chronic)	Androgen/Notch/KDM5 control of fibrosis
(Park et al., 2023) [51]	Mouse; male	Chow or HFD, plus acute/chronic-binge paradigms	Single 5–7 g/kg binge or chronic-plus-binge	Up to 9 h post-binge (Mixed)	ER stress, adipocyte death, FAEEs
(Schonfeld et al., 2021) [52]	Mouse; both sexes	Western diet (40% calories from fat)	10% or alternating 20/10% ethanol in water	16 weeks (Chronic)	WDA model development, fibrosis, HNF4α dysfunction
(Schonfeld et al., 2022b) [53]	Mouse; both sexes	Lieber-DeCarli and WDA	4.8% liquid diet or 20/10% WDA	3-16 weeks (Chronic)	Male-specific KDM5B/KDM5C injury and dedifferentiation
(Schonfeld et al., 2022a) [54]	Mouse; both sexes	Lieber-DeCarli and WDA	4.8% liquid diet or 20/10% WDA	3-16 weeks (Chronic)	Female-specific KDM5B/KDM5C fibrosis and AhR signalling
(Schonfeld et al., 2025) [55]	Mouse; both sexes	WDA	Ramped drinking-water ethanol to 20%	16 weeks (Chronic)	Hepatocyte C/EBPβ, HDL remodelling, and fibrosis
(Sengupta et al., 2021) [56]	Mouse; male	WASH/ HFFC high-fat-fructose-cholesterol paradigms	4.5% v/v liquid-diet ethanol	4-7 weeks (Chronic)	Rapid severe steatohepatitis and fibrosis
(Sharma et al., 2023) [57]	Mouse; male	Fast-food diet plus fructose water	Intermittent escalating oral ethanol	8 weeks (Chronic)	NASH-like steatohepatitis, fibrosis, oxidative damage
(Sikhwal et al., 2025) [58]	Mouse; male	HFD + 10% ethanol	Ad libitum 10% ethanol	13-14 weeks (Chronic)	Preference-stratified multi-organ transcriptomics
(Singh et al., 2022) [59]	Mouse; male	HFD	Escalating drinking-water ethanol	12 weeks (Chronic)	Neurobehavior, hippocampal inflammation, liver injury
(Wang et al., 2024) [60]	Mouse; mixed sex in AS, male in AH	HFD single-binge and chronic-plus-binge liquid-diet models	5 g/kg binge; 6.7% liquid-diet ethanol in AH	Acute 9 h endpoints (Acute)	Glucocorticoid receptor control of steatosis and injury
(Wang et al., 2023) [61]	Mouse; male	HFD	Single or repeated 5 g/kg binges	8-12 weeks; 9 h endpoint (Mixed)	Macrophage polarisation, NF-κB/NLRP3
(Willemse et al., 2023) [62]	Mouse; male	Ketogenic vs normal diet	5% then 10% drinking-water ethanol	~3 weeks (Chronic)	mTOR activity and systemic metabolomics
(Xie et al., 2024) [63]	Rat; male	High-sugar/high-fat diet	Ethanol in drinking fluid	4 weeks (Chronic)	Metabolic hypertension, aortic remodelling, RNA-seq
(Yeh et al., 2023) [64]	Mouse; male	Western diet or HFD pre-feeding	Single 5 g/kg binge	3 weeks diet + 9 h endpoint (Mixed)	Severe acute liver injury on a Western diet
(Zhu et al., 2022) [65]	Mouse; male	HFD	Repeated 2.0 g/kg oral ethanol gavage	8 weeks (Chronic)	Colonic injury, toxins, dysbiosis, TLR4/NF-κB

Abbreviations: AhR, aryl hydrocarbon receptor; ApoE-/-, apolipoprotein E knockout; AS, acute steatosis; AH, alcoholic hepatitis; C/EBPβ, CCAAT/enhancer-binding protein beta; CNS, central nervous system; ER, endoplasmic reticulum; FAEEs, fatty acid ethyl esters; HDL, high-density lipoprotein; HFD, high-fat diet; HFFC, high-fat, fructose, and cholesterol; HNF4α, hepatocyte nuclear factor 4 alpha; KDM5B, lysine demethylase 5B; KDM5C, lysine demethylase 5C; LFD, low-fat diet; miR-29a, microRNA-29a; mTOR, mechanistic target of rapamycin; NASH, non-alcoholic steatohepatitis; NF-κB, nuclear factor kappa B; NLRP3, NOD-like receptor family pyrin domain containing 3; RNA-seq, RNA sequencing; TLR4, toll-like receptor 4; v/v, volume/volume; WASH, Western alcohol steatohepatitis; WDA, Western diet alcohol.

Although the review aimed to capture a broad range of dietary patterns, the included evidence base was dominated by obesogenic dietary models, particularly high-fat, Western/fast-food, and related fat-, fructose-, cholesterol-, or sucrose-enriched diets. Only isolated studies evaluated other patterns, including a ketogenic diet. Ethanol exposure was most commonly delivered as chronic continuous administration, with fewer studies using intermittent/repeated binge, single-binge acute, or mixed chronic-plus-binge paradigms. Although several studies used related WDA, HFD, or Lieber-DeCarli model frameworks, no overlapping animal cohorts or duplicate datasets were identified; each report represented an independent experiment.

For synthesis, studies were grouped by exposure duration and pattern. Acute models were defined as single-binge or one-time ethanol exposure with outcomes assessed within hours, typically at approximately 9 h after dosing. Chronic models were defined as repeated or continuous ethanol exposure over weeks, with outcomes measured at the study endpoint. Chronic-plus-binge and repeated-binge studies were considered separately in narrative interpretation when relevant but were not combined with acute single-binge models.

Most studies focused on hepatic and circulating outcomes. Liver-related endpoints and circulating biomarkers predominated, whereas extrahepatic mechanistic outcomes were less frequently assessed and included gut/microbiome, skeletal muscle, brain/CNS, adipose, and vascular/cardiometabolic tissues. Detailed outcome-level extraction is provided in S1 Dataset in S1 File.

### Risk of bias in studies

Risk-of-bias assessments are summarised in Fig 2, with complete study-level and domain-level judgments provided in S2 Table in S1 File. Overall, most studies were judged as low or unclear risk across domains, with uncertainty driven primarily by incomplete reporting rather than consistently identified major methodological flaws. Low-risk judgments were most frequent for baseline characteristics, incomplete outcome data, random housing and selective outcome reporting, indicating that most studies adequately described group comparability at baseline and reported outcome data without obvious evidence of attrition-related or reporting-related bias. By contrast, allocation concealment, blinding of investigators, random outcome assessment, and blinding of outcome assessment were most often rated as unclear, reflecting limited methodological detail in the primary reports rather than explicit evidence of poor practice. This thereby reduces confidence in the direction and magnitude of the observed effects.

**Fig 2:**
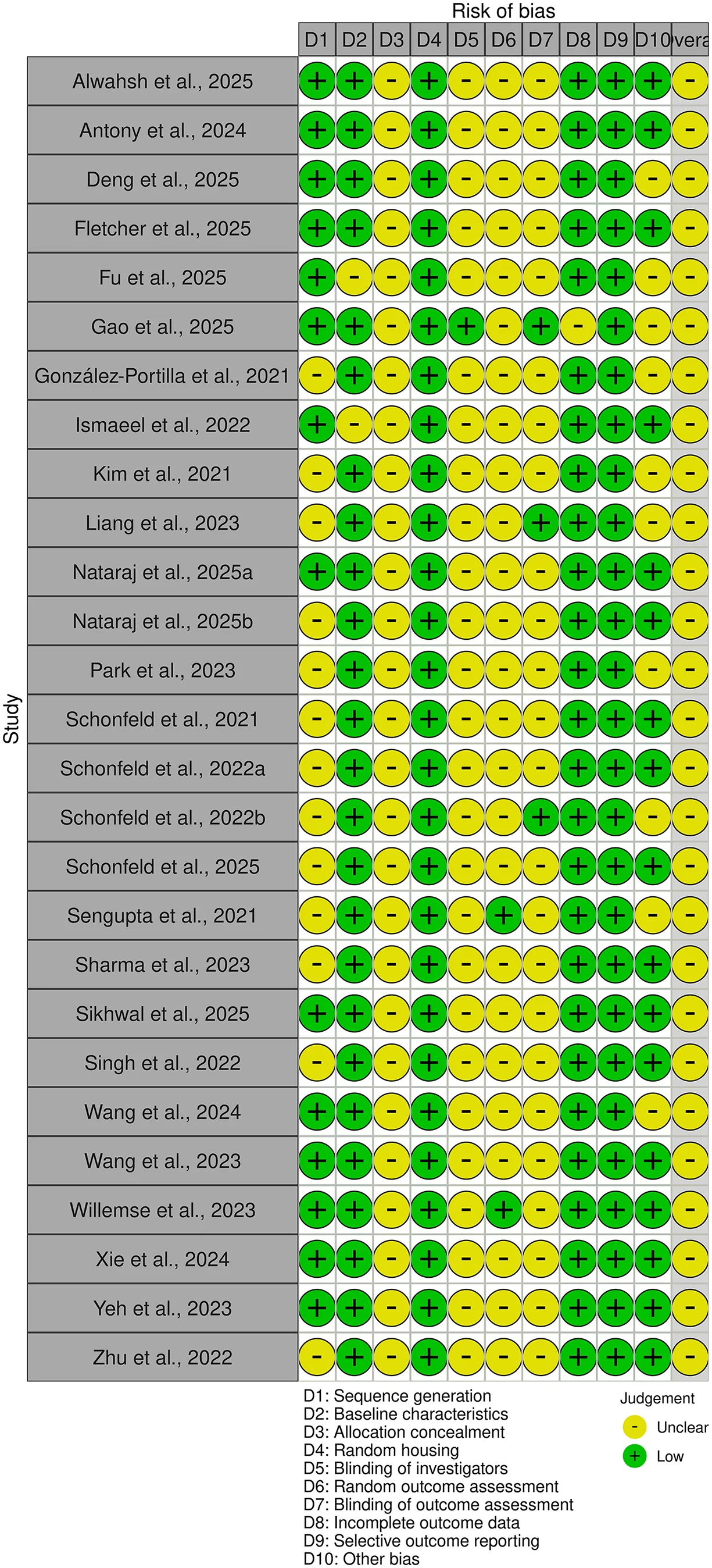
Risk-of-bias assessment of included preclinical studies. Study-level risk of bias was assessed using the SYRCLE risk-of-bias tool. Green symbols indicate low risk of bias, yellow symbols indicate unclear risk of bias, and red symbols indicate high risk of bias.

### Results of individual studies

Detailed outcome-level findings for each study are presented in S1 Dataset in S1 File. Across studies, the most consistent individual-study findings were observed in the liver. Combined diet-ethanol exposure generally produced greater hepatocellular injury, steatosis, and lipid dysregulation than diet-only or ethanol-only conditions, particularly in acute binge-on-diet models and chronic obesogenic diet models [39,41,47,51,56,57,59–61,64]. Chronic Western/WDA and fast-food-type models additionally showed more advanced fibrotic remodelling, extracellular matrix deposition, and impaired recovery signatures, often linked to transcriptional or epigenetic regulators [40,49,50,52–57]. In parallel, many studies reported accompanying changes in inflammatory, oxidative-stress, ER-stress, and macrophage-polarisation pathways, indicating that worsening of liver phenotype was typically accompanied by broader mechanistic disruption rather than isolated biochemical change [41,47,51,57,59,61,63,65].

Extrahepatic evidence was limited and heterogeneous, providing hypothesis-generating signals rather than firm evidence of a multisystem effect of combined exposure. Gut-focused studies described dysbiosis, faecal metabolomic disturbance, toxin-related pathways, and colonic inflammatory injury [44,58,65]; skeletal-muscle studies reported mitochondrial and redox abnormalities [42,46,58]; brain/CNS studies identified neuroinflammatory and behavioral alterations [45,58,59]; and vascular studies suggested adverse cardiometabolic remodelling [43,63]. A subset of studies contributed proteomic, transcriptomic, metabolomic, or epigenetic analyses that added mechanistic depth, but these remained concentrated within obesogenic diet models and were not sufficiently comparable for quantitative pooling [40,43,44,48–50,55,58,62,63]. The study-level evidence suggests that the retrieved literature supports a model in which obesogenic dietary context modifies the severity and mechanistic profile of ethanol-induced injury, while evidence for non-obesogenic dietary patterns remains sparse. Notably, the single ketogenic diet study did not primarily show the steatotic injury pattern typical of obesogenic models, but instead reported changes in mTOR activity, systemic metabolism, redox balance, and a possible attenuation of collagen degradation during chronic alcohol exposure, pointing to potentially distinct mechanisms in non-obesogenic dietary settings [62].

### Results of syntheses

#### Summary of studies contributing to each synthesis.

Quantitative synthesis was feasible only for recurrent hepatic outcomes with sufficiently extractable summary data. Acute and chronic models were analysed separately because they reflect biologically distinct exposure contexts. Outcomes were pooled using standardised mean differences because reporting units varied across studies. Fibrosis-related measures were not pooled because scales and morphometric readouts were insufficiently harmonised across models.

#### Narrative synthesis.

Across the evidence base, the clearest pattern was that obesogenic dietary environments were associated with more severe ethanol-related injury than lean or standard-diet comparators [39,41,47,51,56,57,59–61,64]. This was most evident for hepatic steatosis, inflammatory activation, and injury markers, and was especially apparent when the diet had already induced a metabolically stressed or steatotic tissue state before ethanol exposure [41,51,60,61,64].

Phenotype also varied by exposure duration. Acute diet-plus-binge models were characterized primarily by abrupt hepatocellular injury, oxidative stress, and lipid accumulation [41,51,60,64], whereas chronic and chronic-plus-binge models more often showed persistent steatosis, immune-cell reprogramming, fibrosis, and transcriptional remodelling [40,49,50,52–57]. Although extrahepatic evidence remained limited, findings across gut, muscle, brain, and vasculature suggests a multisystem model in which dietary context could shape both the severity and mechanistic profile of ethanol-induced injury [42–46,58,59,63,65].

#### Meta-analyses of acute models.

Pairwise meta-analyses were first performed to assess the effect of adding ethanol to an HFD background in acute models. Compared with HFD alone, HFD + EtOH significantly increased serum ALT (SMD 2.31, 95% CI 1.46 to 3.16; I² = 13%) and serum AST (SMD 1.18, 95% CI 0.54 to 1.81; I² = 0%), indicating greater acute hepatocellular injury following ethanol exposure in HFD-fed animals (Fig 3a and 3b). Hepatic triglycerides were also significantly higher in the HFD + EtOH group (SMD 2.06, 95% CI 0.96 to 3.17; I² = 52%), consistent with increased intrahepatic lipid accumulation (Fig 3d). Serum triglycerides were higher on average in the HFD + EtOH group, but the pooled difference did not reach statistical significance (SMD 1.22, 95% CI −0.10 to 2.54; I² = 75%; P = 0.07), with substantial between-study heterogeneity (Fig 3c).

**Fig 3:**
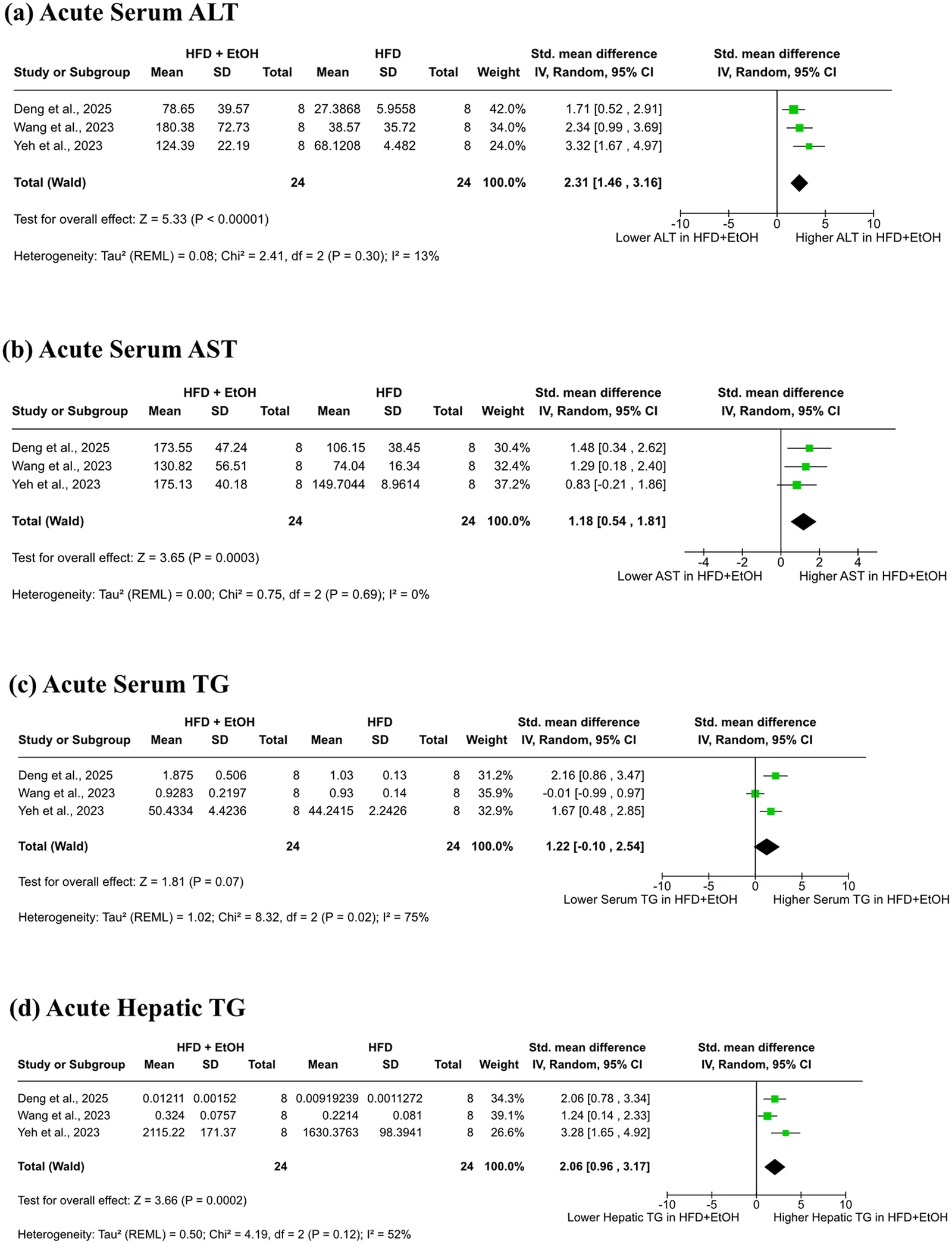
Acute pairwise meta-analyses of ethanol-associated liver injury and triglyceride outcomes in high-fat diet-fed animals. Forest plots showing standardised mean differences comparing high-fat diet plus ethanol (HFD + EtOH) with HFD-only groups for (a) serum alanine aminotransferase (ALT), (b) serum aspartate aminotransferase (AST), (c) serum triglycerides (TG), and (d) hepatic triglycerides in acute ethanol-exposure models. Positive estimates indicate higher outcome values in the HFD + EtOH group, whereas negative estimates indicate lower values in the HFD + EtOH group. Squares represent individual study estimates, with square size proportional to study weight; horizontal lines represent 95% confidence intervals (CIs), and diamonds represent pooled estimates and their 95% CIs. Estimates were pooled using inverse-variance random-effects models. CIs were calculated using the Wald-type method. Tau² was calculated using the restricted maximum-likelihood (REML) method. SMD, standardised mean difference; HFD, high-fat diet; EtOH, ethanol; ALT, alanine aminotransferase; AST, aspartate aminotransferase; TG, triglycerides.

Formal interaction analyses were feasible for two acute studies with complete 2 × 2 designs comprising control, ethanol-only, diet-only, and diet-plus-ethanol groups. The pooled interaction effect for serum ALT was positive and statistically significant (MD 74.56, 95% CI 59.03 to 90.10; I² = 7%), indicating a greater ethanol-associated increase in ALT under high-fat dietary exposure (Fig 4a). For serum AST, the pooled interaction effect was also positive but not statistically significant, with considerable between-study heterogeneity (MD 114.64, 95% CI −36.08 to 265.36; I² = 97%) (Fig 4b). Serum triglycerides showed a negative, non-significant interaction effect (MD −11.98, 95% CI −32.59 to 8.64; I² = 57%), suggesting a tendency toward a smaller ethanol-associated increase in the high-fat dietary context (Fig 4c). Hepatic triglycerides showed a positive but non-significant interaction effect (MD 108.03, 95% CI −47.97 to 264.02; I² = 69%) (Fig 4d).

**Fig 4:**
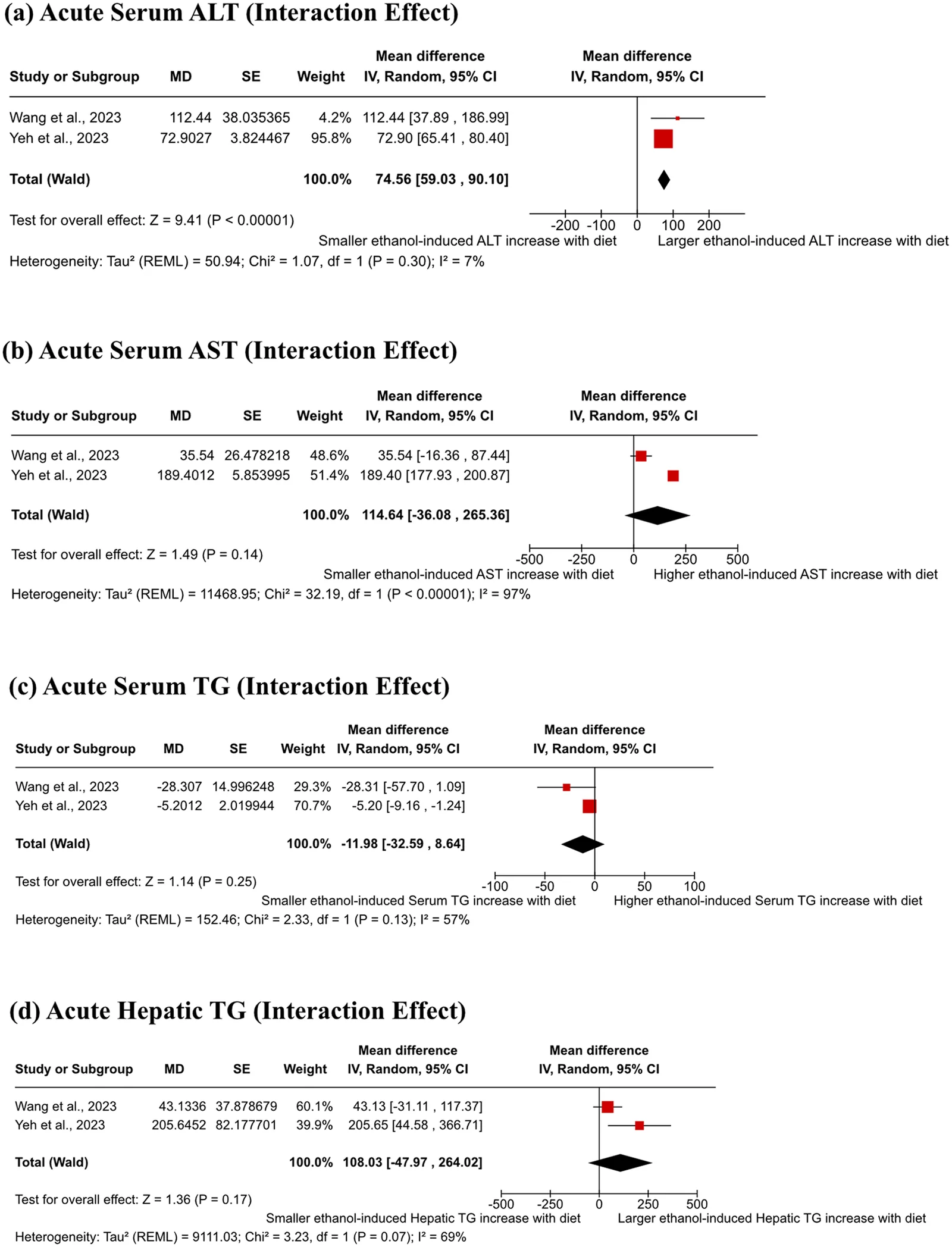
Acute interaction meta-analyses of dietary context on ethanol-induced liver injury and triglyceride outcomes. Forest plots showing interaction mean differences assessing whether high-fat dietary exposure modified ethanol-associated changes in (a) serum alanine aminotransferase (ALT), (b) serum aspartate aminotransferase (AST), (c) serum triglycerides (TG), and (d) hepatic triglycerides in acute models. Positive interaction estimates indicate a larger ethanol-induced increase in the high-fat dietary context, whereas negative estimates indicate a smaller ethanol-induced increase. Squares represent individual study estimates, with square size proportional to study weight; horizontal lines represent 95% confidence intervals (CIs), and diamonds represent pooled estimates and their 95% CIs. Estimates were pooled using inverse-variance random-effects models. CI calculated using the Wald-type method. Tau² calculated using the restricted maximum-likelihood (REML) method. MD, mean difference; SE, standard error.

#### Meta-analyses of chronic models.

Pairwise meta-analyses were performed to assess the effect of adding ethanol to a dietary-exposure background in chronic models. Across seven studies, serum ALT was higher in the diet-plus-ethanol group than in the diet-only group, although the pooled effect was not statistically significant and substantial between-study heterogeneity was present (SMD 1.07, 95% CI −0.43 to 2.57; I² = 90%) (Fig 5a). Serum AST also showed a positive but non-significant pooled effect, with considerable heterogeneity across the seven studies (SMD 0.78, 95% CI −0.09 to 1.64; I² = 67%) (Fig 5b). Similarly, hepatic triglycerides tended to be higher in the diet-plus-ethanol group across three studies, but the pooled effect did not reach statistical significance and heterogeneity was substantial (SMD 1.51, 95% CI −0.17 to 3.18; I² = 75%) (Fig 5c).

**Fig 5:**
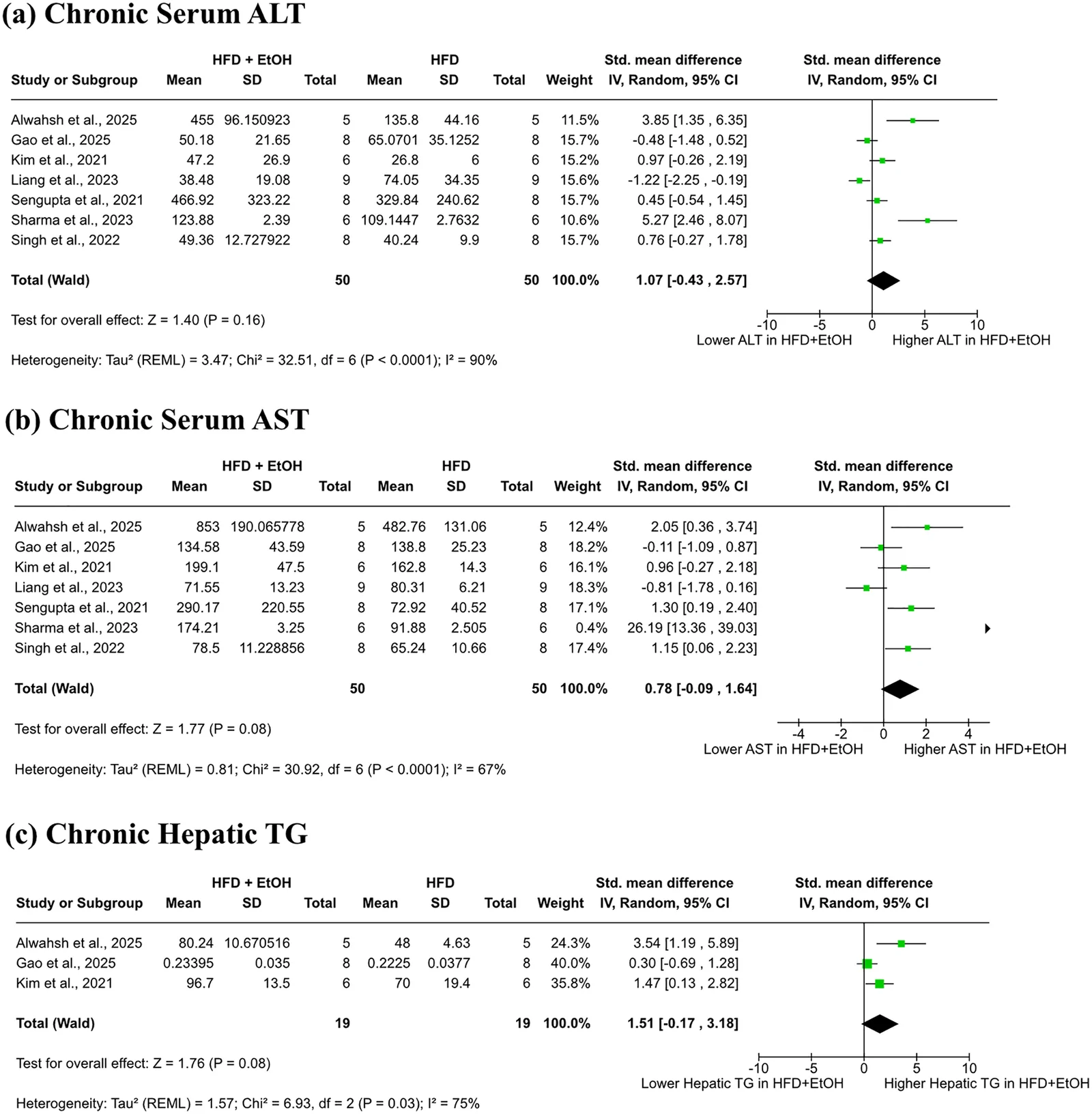
Chronic pairwise meta-analyses of ethanol-associated liver injury and hepatic triglyceride accumulation in diet-fed animals. Forest plots showing standardised mean differences comparing diet-plus-ethanol with diet-only groups for (a) serum alanine aminotransferase (ALT), (b) serum aspartate aminotransferase (AST), and (c) hepatic triglycerides (TG) in chronic ethanol-exposure models. Positive estimates indicate higher outcome values in the diet-plus-ethanol group, whereas negative estimates indicate lower values in the diet-plus-ethanol group. Squares represent individual study estimates, with square size proportional to study weight; horizontal lines represent 95% confidence intervals (CIs), and diamonds represent pooled estimates and their 95% CIs. Estimates were pooled using inverse-variance random-effects models. CIs were calculated using the Wald-type method. Tau² was calculated using the restricted maximum-likelihood (REML) method. SMD, standardised mean difference; ALT, alanine aminotransferase; AST, aspartate aminotransferase; TG, triglycerides.

Formal interaction analyses were then performed in chronic studies with complete 2 × 2 group structures. For serum ALT, the pooled interaction effect was close to null and not statistically significant (MD −1.56, 95% CI −4.75 to 1.63; I² = 0%) (Fig 6a). Similarly, the pooled interaction effect for serum AST was not statistically significant, although substantial between-study heterogeneity was observed (MD 18.64, 95% CI −31.23 to 68.52; I² = 83%) (Fig 6b). In contrast, the pooled interaction effect for hepatic triglycerides was negative and statistically significant (MD −0.05, 95% CI −0.09 to −0.00; I² = 0%), indicating a smaller ethanol-associated increase in hepatic triglycerides in the dietary-exposure context (Fig 6c).

**Fig 6:**
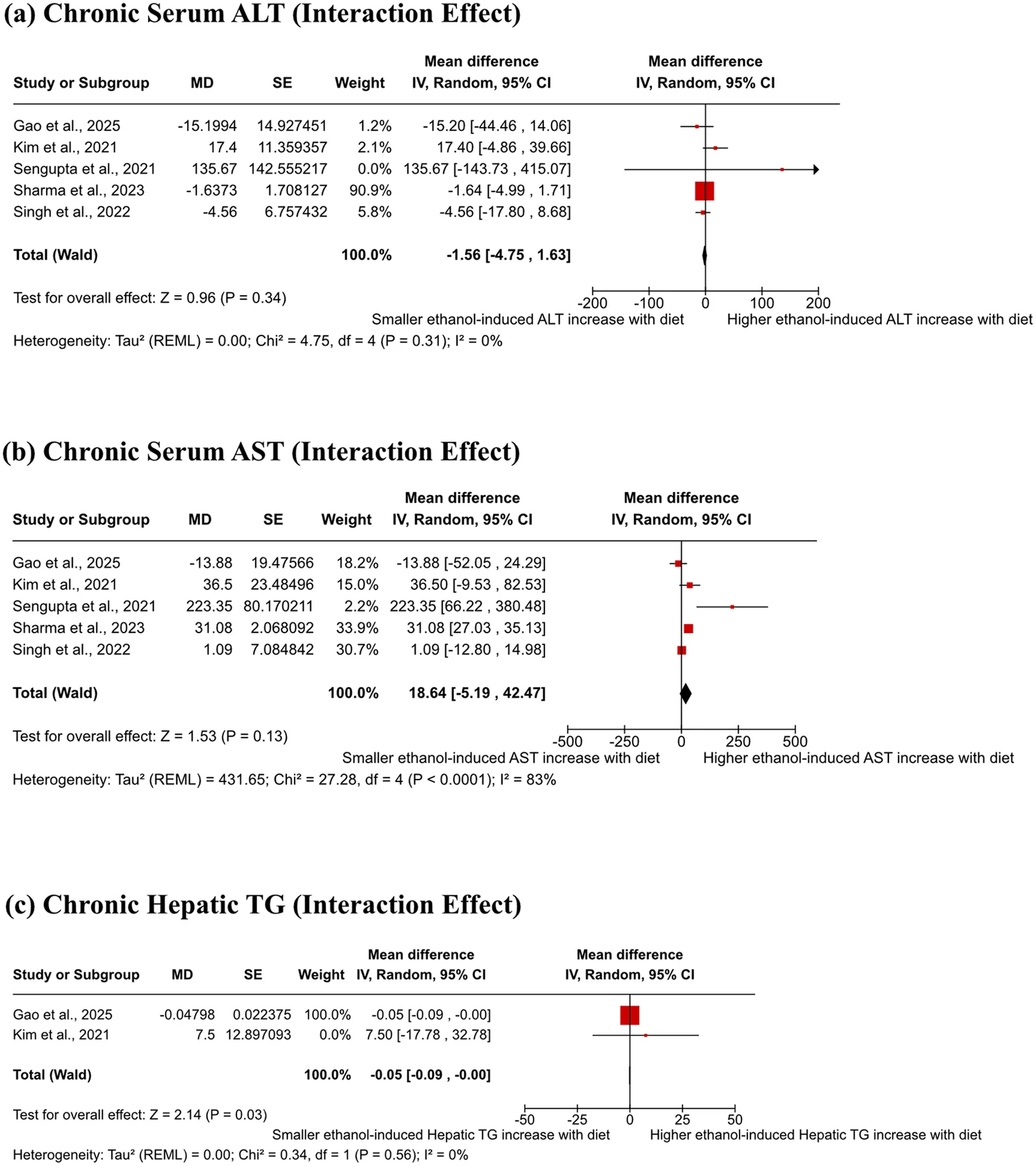
Chronic interaction meta-analyses of dietary context on ethanol-induced liver injury and hepatic triglyceride accumulation. Forest plots showing interaction mean differences assessing whether dietary exposure modified ethanol-associated changes in (a) serum alanine aminotransferase (ALT), (b) serum aspartate aminotransferase (AST), and (c) hepatic triglycerides (TG) in chronic models. Positive interaction estimates indicate a larger ethanol-induced increase in the dietary-exposure context, whereas negative estimates indicate a smaller ethanol-induced increase. Squares represent individual study estimates, with square size proportional to study weight; horizontal lines represent 95% confidence intervals (CIs), and diamonds represent pooled estimates and their 95% CIs. Estimates were pooled using inverse-variance random-effects models. CIs were calculated using the Wald-type method. Tau² was calculated using the restricted maximum-likelihood (REML) method. MD, mean difference; SE, standard error.

## Discussion

The contemporary evidence base was predominantly composed of obesogenic dietary models and was strongly liver-dominant. Although some studies assessed gut, skeletal-muscle, brain, adipose, and vascular outcomes, these organ systems were represented by substantially fewer and more heterogeneous studies than the liver. Evidence concerning non-obesogenic dietary patterns was particularly limited, with only one included study evaluating a ketogenic diet. These features, together with the predominance of studies using mice and male animals and variation in ethanol exposure paradigms, limit the generalisability of the findings.

Across the included studies, the most consistent finding was that obesogenic dietary conditions were associated with greater ethanol-related liver injury in preclinical models, particularly steatosis, inflammatory activation, oxidative stress, and, with sustained exposure, fibrotic remodelling. These observations are compatible with the hypothesis that pre-existing metabolic stress increases susceptibility to ethanol-related injury, but they do not establish that dietary context alters a general biological threshold at which ethanol becomes pathogenic. This hypothesis is relevant to the emerging MetALD framework, although the interaction evidence was outcome- and duration-specific and did not demonstrate uniform synergy between metabolic dysfunction and alcohol [66,67]. The single ketogenic diet study reported a different pattern involving mTOR signalling, systemic metabolism, redox balance, and collagen degradation; however, one study is insufficient to define a distinct interaction profile for non-obesogenic diets [62]. Thus, dietary context may influence the severity and mechanistic features of ethanol-related injury in certain preclinical models, but this possibility requires confirmation across a broader range of dietary patterns and organ systems.

The liver emerged as the clearest site of interaction between diet and ethanol exposure. The acute pairwise meta-analysis supports the interpretation that an already metabolically stressed liver is particularly susceptible to ethanol-related hepatocellular injury and lipid accumulation. The acute interaction meta-analysis, however, provided evidence of dietary effect modification principally for ALT. The absence of consistent interaction effects for AST and triglyceride outcomes suggests that the greater injury observed under combined exposure should not be interpreted as uniform synergy across all endpoints. The differing aminotransferase patterns may partly reflect the greater liver specificity of ALT, whereas AST has substantial extrahepatic sources, including skeletal muscle [68]. One possible explanation is that ethanol and obesogenic diets converge on pathways involving substrate overload, impaired mitochondrial quality control, redox imbalance, and inflammatory signalling [41,47,57]. The positive point estimates for both hepatic and circulating triglycerides are consistent with lipid dysregulation under combined diet and ethanol exposure [69]. However, the serum triglyceride estimate did not reach statistical significance and showed substantial heterogeneity, so the circulating response remains uncertain. Several included studies also reported alterations in oxidative stress, ER stress, inflammasome activation, pyroptotic signalling, and macrophage-driven inflammatory responses [51,59,61,65]. These pathways represent candidate contributors to the observed injury, although their causal and mediating roles require direct functional testing.

In chronic models, the phenotype shifted from acute biochemical injury toward persistent tissue remodelling and progressive disease signatures. Ethanol superimposed on obesogenic diets was associated with sustained steatosis, inflammatory activation, fibrosis-related changes, extracellular matrix deposition, and molecular signatures linked to disease progression and hepatocellular dedifferentiation [40,49,50,52–54,56,57]. The chronic pairwise meta-analysis provided less consistent support for a uniform worsening of biochemical outcomes, suggesting that the effects of combined exposure become more variable with prolonged treatment. Similarly, the chronic interaction meta-analysis did not support consistent positive effect modification across outcomes. In particular, the inverse interaction pattern for hepatic triglycerides suggests that pre-existing dietary lipid accumulation, compensatory adaptation, or ceiling effects may influence the additional response to ethanol rather than indicating a protective effect of diet. This likely reflects the increasing biological complexity of prolonged exposure states, in which tissue phenotype is shaped not only by injury amplification, but also by immune-cell reprogramming, stellate-cell activation, extracellular matrix remodelling, and failed repair processes [70,71]. Thus, chronic diet-plus-ethanol exposure may contribute to a progressively unstable disease state, although the magnitude and nature of this effect appear to depend on the outcome and experimental context.

A particularly important theme in the chronic studies was the emergence of transcriptional, proteomic, metabolomic, and epigenetic dysregulation. These findings identify candidate regulatory pathways through which dietary conditions could influence ethanol-related tissue injury and repair [40,48,49,55,58]. The observed associations raise the possibility of alterations in hepatocyte identity, inflammatory-state transitions, macrophage phenotype, and fibrogenic signalling, but they do not establish persistent regulatory reprogramming or causal mediation. Functional and time-course studies are therefore needed to determine whether these molecular changes contribute directly to disease progression. The increasing use of transcriptomic and multi-omics approaches nevertheless represents an important transition from descriptive phenotyping toward systems-level mechanistic investigation.

Although hepatic outcomes dominated the included studies, the limited extrahepatic findings raise the possibility that combined dietary and ethanol exposure may affect other organ systems. Gut-focused studies reported alterations in the intestinal barrier, microbiome, and inflammatory pathways, consistent with a potential contribution of the gut-liver axis [44]. Studies involving the brain and skeletal muscle similarly identified changes in mitochondrial and inflammatory pathways. However, the small number and heterogeneity of these studies are insufficient to establish a multisystem diet-ethanol interaction or determine whether the same mechanisms operate across organs [8,22,58,66]. Further studies are required to define the extent and biological significance of these extrahepatic effects.

This review has some limitations that should temper interpretation. First, the restriction to studies published from 2021 onward may have excluded foundational and potentially relevant earlier animal evidence. Accordingly, the findings should be interpreted as a synthesis of contemporary preclinical evidence rather than a cumulative account of the entire field. Second, the included literature was heavily concentrated in mice, male animals, and high-fat or Western-type diets, which limits external validity across species, sexes, life stages, and dietary patterns with potentially neutral or protective effects. Third, ethanol exposure paradigms varied substantially across drinking water, liquid diet, gavage, binge, and chronic-plus-binge designs, and these differences influence blood alcohol kinetics, caloric substitution, stress exposure, and feeding behaviour, making between-study equivalence imperfect even when outcomes appear conceptually similar. Furthermore, extrahepatic, microbiome, and multi-omics outcomes were reported in relatively few studies, so the review could point to multisystem pathways but could not quantify them with the same confidence as hepatic endpoints.

Future research should expand beyond high-fat and Western diets to include more diverse, real-world dietary patterns, while improving the precision and reporting of ethanol exposure to enhance comparability and reproducibility. Studies should incorporate both sexes with clear sex-specific analyses and move beyond liver-focused models to include multisystem outcomes such as gut, brain, muscle, and vascular effects. In addition, omics findings should be supported by functional and time-course validation to strengthen causal inference. Overall, more translationally relevant models are needed to reflect combined metabolic and alcohol-related disease and to support integrated, multi-target intervention strategies.

## Conclusion

The strongest evidence indicates that obesogenic diets, particularly high-fat and Western-type patterns, are associated with more severe ethanol-related hepatic injury in preclinical models, including steatosis, lipid dysregulation, oxidative stress, inflammation, and fibrosis. Extrahepatic findings involving the gut, brain, skeletal muscle, adipose tissue, and vasculature, together with microbiome and multi-omics findings, remain limited, heterogeneous, and suggestive rather than conclusive. Evidence for non-obesogenic diets was limited to a single ketogenic-diet study. Dietary context may therefore modify ethanol toxicity in specific preclinical models, but the predominance of studies using mice and male animals and the heterogeneity of ethanol exposure paradigms limit generalisability.

## Supporting information

S1 File S1 Appendix. Complete database search strings used for PubMed, Embase, and Scopus, including database-specific syntax, limits, and search dates. S1 Table. Detailed characteristics of the included studies. Animal models, dietary and ethanol exposures, comparators, and study-design variables. S1 Dataset. Complete study-level outcome dataset and analysis inputs. Extracted and digitized outcome values, sample sizes, group means and standard deviations, reconstructed or converted values, interaction estimates and standard errors, and the analysis-ready inputs used in RevMan. S2 Table. Risk-of-bias judgments for the included studies. Complete study-level and domain-level assessments using the SYRCLE risk-of-bias tool.
(ZIP)
