# Supplementary material for: Dietary context and ethanol-related injury in preclinical models: A contemporary systematic review of predominantly obesogenic diets and liver outcomes
Source: PLoS One. 2026 Oct 21;21(10):e0352561. doi: 10.1371/journal.pone.0352561 (PMC13686270; doi:10.1371/journal.pone.0352561)
Supplement: Supplementary file 1 [file pone.0352561.s001.zip › Supplementary Files/S1 Appendix.docx]

**S1 Appendix**

**Complete database-specific search strings**

This appendix presents the complete search strings used to identify eligible preclinical studies examining ethanol exposure in the context of defined dietary patterns. PubMed, Embase, and Scopus were searched on 15 January 2026. Each search combined two core concepts: (1) ethanol or alcohol exposure and (2) dietary pattern or composition. Synonymous terms within each concept were combined using “OR,” and the two concept blocks were combined using “AND.” Organ-system and mechanistic terms were not incorporated as additional search blocks; these eligibility criteria were assessed during study screening. The searches were restricted to records published from 1 January 2021 to 15 January 2026.

**Embase**

Database and platform: Embase.com (Elsevier)

Date searched: 15 January 2026

Fields searched: Title, abstract, and author keywords

Publication period: 1 January 2021-15 January 2026

Complete search string:

(Ethanol OR alcohol OR 'Lieber-DeCarli' OR 'alcohol liquid diet' OR 'intragastric ethanol' OR 'ethanol vapor' OR binge OR 'chronic ethanol'),ab,kw AND ('western diet' OR 'high fat diet' OR HFD OR HFHS OR 'cafeteria diet' OR 'high sucrose' OR 'high fructose' OR ketogenic OR 'ketogenic diet' OR 'low carbohydrate' OR carbohydrate OR 'very low carbohydrate' OR 'Mediterranean diet' OR 'olive oil'),ab,kw

Limits applied: Publication date, 1 January 2021-15 January 2026

**PubMed**

Database and platform: PubMed, National Center for Biotechnology Information, US National Library of Medicine

Date searched: 15 January 2026

Fields searched: Title and abstract

Publication period: 1 January 2021-15 January 2026

Complete search strategy:

(Ethanol[tiab] OR alcohol[tiab] OR "Lieber-DeCarli"[tiab] OR "alcohol liquid diet"[tiab] OR "intragastric ethanol"[tiab] OR "ethanol vapor"[tiab] OR binge[tiab] OR "chronic ethanol"[tiab]) AND ("western diet"[tiab] OR "high fat diet"[tiab] OR HFD[tiab] OR HFHS[tiab] OR "cafeteria diet"[tiab] OR "high sucrose"[tiab] OR "high fructose"[tiab] OR ketogenic[tiab] OR "ketogenic diet"[tiab] OR "low carbohydrate"[tiab] OR "carbohydrate"[tiab] OR "very low carbohydrate"[tiab] OR "Mediterranean diet"[tiab] OR "olive oil"[tiab])

Limits applied: Publication date, 1 January 2021-15 January 2026

**Scopus**

Database and platform: Scopus (Elsevier)

Date searched: 15 January 2026

Fields searched: Title, abstract, and keywords

Publication period: 1 January 2021-15 January 2026

Complete search strategy:

TITLE-ABS-KEY(Ethanol OR alcohol OR "Lieber-DeCarli" OR "alcohol liquid diet" OR "intragastric ethanol" OR "ethanol vapor" OR binge OR "chronic ethanol") AND TITLE-ABS-KEY("western diet" OR "high fat diet" OR HFD OR HFHS OR "cafeteria diet" OR "high sucrose" OR "high fructose" OR ketogenic OR "ketogenic diet" OR "low carbohydrate" OR "carbohydrate" OR "very low carbohydrate" OR "Mediterranean diet" OR "olive oil")

Limits applied: Publication date, 1 January 2021-15 January 2026
